# Artificial Intelligence in Oncology: A Topical Collection in 2022

**DOI:** 10.3390/cancers15041065

**Published:** 2023-02-07

**Authors:** Andreas Stadlbauer, Anke Meyer-Bäse

**Affiliations:** 1 Institute of Medical Radiology, University Clinic St. Pölten, Karl Landsteiner University of Health Sciences, 3100 St. Pölten, Austria; 2 Department of Neurosurgery, Friedrich-Alexander University (FAU) Erlangen-Nürnberg, 91054 Erlangen, Germany; 3 Department of Scientific Computing, Florida State University, 400 Dirac Science Library, Tallahassee, FL 32306-4120, USA

Artificial intelligence (AI) is considered one of the core technologies of the Fourth Industrial Revolution that is currently taking place. These approaches bridge the gap between collecting data and interpreting them meaningfully, and have demonstrated outstanding capabilities that have surpassed most previous classification and regression methods. In addition to a variety of industrial applications, AI has made significant contributions to solving a variety of biomedical problems, including cancer, over the past decade.

Deep learning (DL), a branch of machine learning (ML) and AI, is based on artificial neural networks but differs from them in the depth and number of hidden layers. Due to its ability to learn from data, DL technology is increasingly used in various applications of daily use such as visual recognition, text analysis and translation, cyber security or image and text generation, but, of course, also in healthcare.

AI, and in particular DL, can play a helpful role in a variety of aspects of oncology: tumor detection segmentation and classification, histopathological diagnosis, tracking of tumor development, clinical decision making and validation of cancer therapies or prognosis predictions. In oncology, there is a strong need for tools that can improve both the routines of clinicians and patient care. In a decision-making area as crucial as healthcare, transparency and reliability are essential to gain the trust of physicians and patients. The scientific community therefore has the task of examining the application of AI algorithms with regard to the high requirements for medical applications.

This topical collection, which has been running since the beginning of 2021, has thus far included a total of 44 articles. In 2022, 22 articles (18 original articles and 4 reviews) were published in it. The range of topics is broad, and the articles deal with the most diverse aspects of the state of the art and future perspectives in the field of clinical research on the application of “Artificial Intelligence in Oncology”. Of the 18 original articles, 7 dealt with the application of ML and DL to classify different aspects of tumors, followed by 6 articles on prediction. The remaining five articles dealt with image augmentation, quality assessment, data extraction, tumor detection, and screening.

Two reviews gave an overview of new developments in the use of DL in computational histopathology. The great success of DL technology in the field of computer vision has boosted the considerable interest in digital pathology analysis. In their review, Wu et al. [1] aimed to provide a comprehensive and up-to-date review of the deep learning methods for digital H&E-stained pathology image analysis, including color normalization, nuclei/tissue segmentation, and cancer diagnosis and prognosis. They also provided online resources and discussed open research problems in pathological image analysis. They concluded that the existing studies demonstrated that deep learning is a promising tool to assist clinicians in the clinical management of human cancers. The second review focused on a comprehensive summary of algorithms for AI-supported detection and classification of gastrointestinal cancers [2]. Wong et al. provided an overview of the current histological practices in anatomical pathology laboratories with respect to challenges faced in image preprocessing, presented the existing AI-based algorithms, discussed their limitations and presented clinical insight with respect to the application of AI in the early detection and diagnosis of gastrointestinal cancer.

One review addressed the role of ML in improving screening. Screening and early detection of cancer are key factors in reducing the burden of this disease. Conventional diagnostic approaches have made little use of the vast amounts of clinical and diagnostic data that are routinely being collected along the diagnostic pathway. Computational and “intelligent” support is required to handle this ever-increasing flood of data. Benning et al. [3] gave an overview of the theoretical foundations of ML approaches and elaborated on the established and potential use cases of ML algorithms in screening and early detection. They discussed the relevant challenges and misconceptions of the applicability of ML-based diagnostic approaches and emphasized the need for a clear regulatory framework to responsibly introduce ML-based screening and early detection approaches in the clinical routine. They concluded that ML has the potential to become another important driver in addressing the global burden of cancer if scientists and practitioners consider identifying and assembling appropriate input data within an ethical and regulatory framework to ensure data safety and security.

ML techniques also offer a promising solution for efficiently extracting clinically relevant information from unstructured text found in patient documents. This was the subject of the fourth review by Estevez et al. [4]. The extraction of real-world data (e.g., pathology reports and clinical notes) from unstructured text in electronic health records by humans is both difficult and costly. The remarkable advances in natural language processing and ML techniques provide promising solutions for a variety of information extraction tasks. However, this also poses particular challenges in terms of assessing validity and generalizability to different cohorts of interest. The authors proposed a research-centric evaluation framework for model developers, ML-extracted data users and other real-world data stakeholders in order to enable effective and accurate use of ML to extract real-world data.

Two of the seven original articles on classification dealt with the diagnosis of ovarian cancer. Saida et al. [5] explored the clinical applicability of a convolutional neural network (CNN) as a reading assist for the differentiation of ovarian carcinomas and borderline tumors on MRI, and compared the diagnostic performance with interpretations by experienced radiologists. The CNN in combination with maps of the apparent diffusion coefficient (ADC) showed the highest diagnostic performance among all sequences including T2-weighted and contrast-enhanced T1-weighted MRI that was equivalent to that of the radiologists. They concluded that, although diagnostic imaging of ovarian tumors is complex, deep learning has shown good diagnostic performance for diagnosing ovarian carcinomas, including borderline tumors.

The pilot study of van Vliet-Perez et al. [6] evaluated the feasibility of near-infrared hyperspectral imaging to detect ovarian cancer in images of surgically removed tissue samples. The authors compared the hyperspectral imaging data with histopathology in order to train a linear support vector machine. They were able to classify tumorous tissue with a sensitivity of 0.81 and a specificity of 0.70, and concluded that the technique has the potential to enable real-time image guidance during advanced-stage ovarian cancer surgery in order to intraoperatively detect microscopic tumors, which would be of great value.

Two publications from the group of Kanavati and Tsuneki evaluated the utility of DL for the classification of whole-slide images. Kanavati et al. [7] used of a DL model composed of a CNN and a recurrent neural network (RNN) for the classification of whole-slide images of liquid-based cytology specimens into neoplastic and non-neoplastic for cervical cancer screening. Their ensemble network was trained with 1605 cervical whole-slide images, and the model was evaluated on three test sets with a combined total of 1468 cervical whole-slide images. They achieved an area under the receiver operating characteristic curve (AUROC) in the range of 0.89–0.96 and concluded that it represents a promising potential use of such models for aiding screening processes.

In their second paper [8], they used transfer and weakly supervised learning of a pre-trained DL model (ImageNet) to classify prostate whole-slide images from transurethral resection into prostate adenocarcinoma and benign (non-neoplastic) lesions. The models were evaluated on test sets from transurethral prostate resection, needle biopsy and a public dataset from The Cancer Genome Atlas (TCGA). They achieved an AUROC of up to 0.984 in transurethral prostate resection test sets for adenocarcinoma and concluded on the promising potential of their model in a practical histopathological diagnostic workflow system to improve the efficiency of pathologists.

In two other publications, a preoperative classification of brain tumors using traditional ML algorithms and MRI data was performed. In meningioma patients, a high Ki-67 proliferative index is associated with an increased recurrent risk. The study of Zhao et al. [9] explored the value of radiomic features from contrast-enhanced MRI for the preoperative classification of the Ki-67 index in meningioma patients into low-expressed and high-expressed groups with a threshold of 5% using ML models. Their predictive model showed a high diagnostic performance with an AUROC of 0.837 and an accuracy of 0.810 in an internal test dataset as well as a moderate diagnostic performance with an AUROC of 0.700 and an accuracy of 0.557 in an external test dataset. They concluded from their results that the ML models can efficiently predict the Ki-67 index of meningioma patients to facilitate the therapeutic management.

In the second study on brain tumor classification, Stadlbauer et al. [10] combined nine traditional machine learning algorithms with a physiological MRI technique to investigate the effectiveness for multiclass classification of the five most frequent contrast-enhancing brain tumor entities in a clinical setting. They were able to demonstrate that their ML-based approach named radiophysiomics was superior to both human reading and the ML-based classification of conventional MRI data for several performance parameters, including accuracy and classification error. The authors concluded that radiophysiomics could be helpful in the routine diagnostics of contrast-enhancing brain tumors, but the high expenditure of time and work for data processing associated with traditional ML-based technology requires further automation using deep neural networks.

In the remaining publication on tumor classification, Minami et al. [11] developed a tool to classify the depth of submucosal invasion in early colorectal cancer in endoscopic images from preoperative colonoscopy using DL. A convolutional neural network was trained with 706 endoscopic images from 91 patients. The diagnostic accuracy of the CNN was evaluated using 394 images from 49 patients and found to be as high as that of a skilled endoscopist. The authors concluded that endoscopic image recognition by DL might be able to predict the submucosal invasion depth in early-stage colorectal cancer in clinical practice.

Another important area of application of ML and DL algorithms in oncology is prediction, e.g., of the response to therapies, overall survival or other meaningful clinical parameters.

Umutlu et al. [12] assessed whether multiparametric ^18^F-FDG PET/MRI-based radiomics analysis is able to predict pathological complete response to neoadjuvant chemotherapy in breast cancer patients and hence potentially enhance pretherapeutic patient stratification. They included 73 patients with newly diagnosed, therapy-naïve breast cancer that underwent simultaneous ^18^F-FDG PET/MRI examinations. Radiomic data of both ^18^F-FDG PET and MRI (T2-weighted, ADC, dynamic contrast-enhanced) were evaluated separately and in combination with a support vector machine model and 5-fold cross-validation. They obtained the best results for predicting complete pathological response in the entire cohort with the combination of the radiomic features of ^18^F-FDG PET and all MR sequences, with an AUROC of 0.8. In further subgroup analyses, they obtained an AUROC of 0.94 for predicting complete pathologic response in the HR+/HER2– group by combining PET and all MR data. The authors concluded that ^18^F-FDG PET/MRI enables comprehensive high-quality radiomics analysis for the prediction of complete pathological response to neoadjuvant chemotherapy in breast cancer patients, especially in those with HR+/HER2– receptor status.

Giannini et al. [13] developed and validated a delta-radiomics score to predict the response of individual colorectal cancer liver metastases to first-line treatment based on the combination of 5-fluorouracil, leucovorin and oxaliplatin (FOLFOX) chemotherapy. Radiomic features were computed by subtracting textural features of computed tomography (CT) scans at baseline from those after the first cycle of first-line FOLFOX and used to create four different ML algorithms including a support vector machine, logistic regression, decision tree and random forest. The ML models were tested with an external dataset. They obtained a sensitivity of 85% and a specificity of 92%. They concluded that their delta-radiomics signature approach can reliably predict the response of individual colorectal cancer liver metastases to oxaliplatin-based chemotherapy. Furthermore, lesions predicted by the signature to be poor or non-responders could be further investigated, potentially paving the way to lesion-specific therapies.

Jalalifar et al. [14] investigated the impact of tumor segmentation accuracy on the efficacy of quantitative MRI biomarkers of radiotherapy outcome in patients with brain metastases. They used less accurate but automatically generated tumor outlines and studied the impact on the efficacy of the derived imaging biomarkers for radiotherapy response prediction. They were able to demonstrate that while the effect of tumor delineation accuracy is considerable for automatic contours with low accuracy, imaging biomarkers and prediction models are rather robust to imperfections in the produced tumor masks. The authors concluded that their positive outcome paves the way for adopting high-throughput automatically generated tumor masks for discovering diagnostic and prognostic imaging biomarkers in patients with brain metastases without sacrificing accuracy.

Hou et al. [15] constructed a DL prediction model (DeepSurv) based on CT radiomics and key clinical features to generate a personalized survival curve for individual patients with non-small cell lung cancer. Their results showed a good performance in discriminating high and low risks of survival with C-index values between 0.74 and 0.75, and an AUROC between 0.73 and 0.76, respectively. Furthermore, the authors generated personalized survival curves, which could be intuitively applied for individuals in survival prediction in clinical practice. They concluded that the proposed prediction model could benefit physicians, patients and caregivers in managing non-small cell lung cancer and facilitate personalized medicine.

The study of Gomez Marti et al. [16] investigated and clustered risk factors predicting late recurrence of metastatic breast cancer. They applied a previously validated algorithm named Markov Blanket and Interactive Risk Factor Learner (MBIL) to the electronic health records of a publicly available clinical database of breast cancer. Their algorithm provided an output of both single and interacting risk factors of 5-, 10- and 15-year metastases from the database. The clinical relevance of these interactions based on years to metastasis and the reliance on interactivity between risk factors were individually examined and interpreted. They found that a lower interactivity score was associated with a higher prevalence of variables with an independent influence on long-term metastasis (i.e., HER2, TNEG). Increasing interactivity scores, however, required stronger interactions to define clusters of factors that increased the risk of metastasis (i.e., ER, smoking, race, alcohol usage). They concluded that their approach identified single and interacting risk factors of metastatic breast cancer, many of which were supported by clinical evidence, and recommended the development of further large data studies with different databases to validate the degree to which some of these variables impact metastatic breast cancer in the long term.

In another study, Cheng et al. [17] developed a scalable, non-invasive and robust ML model for predicting the axillary lymph node status in early-stage breast cancer integrating ^18^F-FDG Mammi-PET, Lymph-PET, ultrasound, physical examination and clinical characteristics. Least absolute shrinkage and selection operator (LASSO) regression analysis was used in developing the prediction models. In the next step, the authors developed a nomogram based on this model with the best predictive efficiency and clinical utility. The integrated model showed a high performance in identifying pN0 and pN1 with an AUROC of 0.93 in an external validation set. For the clinical N0 subgroup, the negative predictive value was 97%, and for the clinical N1 subgroup, the positive predictive value was 93%. The authors concluded that the use of an integrated ML model can greatly improve the true positive and true negative rate of identifying clinical axillary lymph node status in early-stage breast cancer.

The articles published in 2022 in the topical collection “Artificial Intelligence in Oncology” also showed that there are interesting applications of ML and DL in oncology beyond classification and prediction.

The study of Brahim et al. [18] was also on the subject of improved diagnosis of breast tumors. The authors proposed an approach for an automatic assessment of breast positioning quality in screening mammography using CNNs in order to ensure an adequate breast positioning quality according to predefined criteria. Their approach was intended to assist radiology technicians in identifying inadequately positioned mammograms in real time, reduce the number of returned patients and improve the efficiency of cancer detection. For each predefined criterion, a specific CNN was separately trained and then combined into an overall system that analyzes whether the breast is well positioned or not, achieving an efficient accuracy of 96.5% for craniocaudal and 93.3% for mediolateral oblique images. They concluded that their approach differs from already available studies and commercial tools by taking into account more useful breast positioning criteria that have to be considered by the expert, and thus providing more holistic assistance.

The purpose of the study of Son et al. [19] was to provide guidelines for collecting chest CT scans to facilitate research on the automation of DL-based lung nodule detection. The authors collected chest CT scans from 515 patients with lung nodules from three hospitals and high-quality lung nodule annotations reviewed by radiologists, and included publicly available data from LUNA16 in their experiments. They achieved a better performance when the model was trained on the collected data rather than publicly available LUNA16 data, albeit with a large amount of data. However, they demonstrated that weight transfer learning from pre-trained open data is very useful when it is difficult to collect large amounts of data, and more than 100 patients are required to obtain a good performance. They concluded that their study offers valuable insights for guiding data collection in future lung nodule studies.

The study by Abazari et al. [20] is also in the field of medical imaging. They developed a multi-scale computational framework to generate synthetic ^18^F-FDG PET images similar to the real ones in different stages of solid tumor growth and angiogenesis. Their framework is based on the bio-physiological phenomena of FDG radiotracer uptake in solid tumors combining a biomathematical model and a generative adversarial network (GAN). They achieved a structural similarity index measure of 0.72 and a peak signal-to-noise ratio of 28.53 for the generated PET sample and the experimental sample. With their results, the authors demonstrated that a combination of biomathematical modeling and GAN-based augmentation models provides a robust framework for the non-invasive and accurate generation of synthetic PET images of solid tumors in different stages.

Torrente et al. [21] developed an AI-based tool for cancer patient data analysis that assists clinicians in identifying the clinical factors associated with a poor prognosis, relapse and survival as well as a prognostic model that stratifies patients by risk. For that purpose, the authors evaluated clinical data from 5275 patients diagnosed with non-small cell lung cancer, breast cancer and non-Hodgkin lymphoma in combination with clinical parameters measured with a wearable device and quality of life questionnaire data. They concluded that their AI tools have potential applications in clinical settings to improve risk stratification, early detection and surveillance management of cancer patients.

Finally, in their paper, Böcking et al. [22] presented an AI-powered microscope-based scanner that is able to identify cancer cells in smears from the oral cavity or in body cavity fluids and to determine the degree of malignancy of prostate cancer. Supervised ML algorithms in combination with image features of stained nuclei as well as their DNA content were used to estimate the degree of malignancy. The overall percentage of correct device-derived diagnoses on oral smears was 91.3%, as compared to 75.0% for conventional, subjective investigation. They concluded that their automated microscope-based scanner is able to identify malignant cells in different types of human specimens with a diagnostic accuracy comparable to that of subjective cytological assessment.

The studies published in the topical collection in 2022 impressively show the range of possible and useful applications of ML and DL in oncology. AI will not only revolutionize work processes in industrial manufacturing, but also, if carefully evaluated, have a major impact in healthcare over the next few years. Gaining the acceptance and trust of clinicians and patients in AI-based applications in the clinical routine will be crucial.
